# Identification and Validation of a m6A‐Related Long Noncoding RNA Prognostic Model in Colorectal Cancer

**DOI:** 10.1111/jcmm.70376

**Published:** 2025-01-27

**Authors:** Peng Jiang, Mingfei Chu, Yu Liang

**Affiliations:** ^1^ Department of Colorectal Surgery Cancer Hospital of China Medical University, Liaoning Cancer Hospital & Institute Shenyang China; ^2^ Department of Surgical Oncology and General Surgery The First Hospital of China Medical University Shenyang China

**Keywords:** bioinformatics analysis, colorectal cancer, LINC00543, m6A‐related lncRNA

## Abstract

Accumulating research indicates that N6‐methyladenosine (m6A) modification plays a pivotal role in colorectal cancer (CRC). Hence, investigating the m6A‐related long noncoding RNAs (lncRNAs) significantly improves therapeutic strategies and prognostic assessments. This study aimed to develop and validate a prognostic model based on m6A‐related lncRNAs to improve the prediction of clinical outcomes and identify potential immunological mechanisms in CRC. We obtained high‐throughput CRC data from The Cancer Genome Atlas to identify a prognostic model based on m6A‐related lncRNAs. Then, the model was constructed and validated through LASSO analysis and Cox regression using R software. The clinical applicability was enhanced by developing a nomogram. We further conducted experiments to reveal the biological function of LINC00543. The prognostic model based on eight m6A‐related lncRNAs exhibited impressive accuracy, achieving an area under the receiver‐operating curve value of 0.753, 0.682 and 0.706 for predictions after 1, 3 and 5 years, respectively. The Kaplan–Meier analysis confirmed the consistency of the model across different pathological characteristics, with a high‐risk group showing a poorer prognosis. Furthermore, the model was linked to immune function, particularly the type I interferon response, through gene set enrichment analysis and experimental validation. Our study presented a m6A‐related lncRNA prognostic model for CRC with potential clinical utility. The model not only provided improved accuracy over traditional staging but also offered insights into the immunological mechanisms of CRC, facilitating personalised medicine approaches.

## 1 Introduction

Colorectal cancer (CRC) is recognised as a significant form of gastrointestinal tumour. Its incidence continues to rise globally, making it the third leading cause of cancer‐related mortality. This upward trend in both prevalence and fatality rates has been observed over successive years [1]. The comprehensive treatment system combining surgery with radiotherapy and chemotherapy, targeted therapy, and immunotherapy has significantly improved the prognosis of patients with CRC in recent years [2]. However, CRC cells have strong metastatic ability, and hence curative surgical treatment is not viable for metastatic CRC. Therefore, identifying specific targets and biomarkers for treatment remains a key issue that needs urgent attention in clinical practice.

A significant breakthrough has been witnessed in the field of CRC treatment with the advent of immune checkpoint inhibitors (ICIs). These therapies have demonstrated exceptional efficacy, particularly for patients exhibiting high microsatellite instability (MSI‐H) or those with defects in the mismatch repair. Consequently, ICIs have become the preferred initial therapeutic approach for patients with metastatic CRC who possess these genetic profiles [3]. However, the success of this therapeutic approach is constrained in individuals with proficient mismatch repair or those exhibiting microsatellite stability [4]. N6‐methyladenosine (m6A) is recognised as the most common epigenetic modification in mRNA [5]. Multiple key proteins are involved in m6A modification, including writers (METTL3/14/16, WTAP, VIRMA, ZC3H13 and RBM15/15B), readers (YTHDC1/2, YTHDF1/2, YTHDF2, HNRNPC, FMR1, LRPPRC, HNRNPA2B1, JGFBP1/2/3 and RBMX) and erasers (FTO and ALKBH5) [6]. Recent studies have confirmed the important role of the key m6A‐related proteins in regulating the sensitivity of CRC to immunotherapy. For instance, Bao et al. [7] indicated that YTHDF1 upregulated the expression of CXCL1 by promoting p65 translation and recruited myeloid‐derived suppressor cells (MDSCs) to inhibit T cells. Indeed, targeting YTHDF1 could enhance the therapeutic effect of anti‐PD‐1 in MSS/MSI‐H CRC. Zhai et al. revealed that ALKBH5 recruited MDSCs and limited the activities of NK and T cells via the m6A–AXIN2–Wnt–DKK1 axis in CRC [8]. Hence, further elucidation of the role and mechanisms of m6A modification in CRC is crucial for advancing immunotherapy.

Long noncoding RNAs (lncRNAs) are a class of noncoding RNAs encompassing molecules longer than 200 nucleotides [9, 10, 11, 12]. These molecules can interact with DNA, RNA, and protein molecules, thereby exerting molecular functions at various levels such as epigenetics, transcription and post‐transcriptional modification [13, 14]. Recent studies have also confirmed a regulatory relationship between lncRNAs and m6A‐related proteins. For instance, Zhou et al. reported that HIF‐1α‐induced lncRNA STEAP3‐AS1 competitively interacted with YTHDF2, leading to the dissociation of YTHDF2 from STEAP3 mRNA and subsequent Wnt signalling activation to support CRC progression [15]. Similarly, Hou et al. [16] demonstrated that LINC00460 was linked to reduced overall survival rates at the 5‐year mark in CRC. LINC00460 worked in conjunction with IGF2BP2 and DHX9 to enhance the stability and levels of HMGA1, thereby facilitating the proliferation, migration, and invasion of CRC cells. Therefore, further exploration of the mechanisms by which lncRNAs regulate m6a‐related genes is crucial for advancing research on immunotherapy for CRC.

Despite substantial advances in understanding m6A modification and its roles in CRC, prior studies have predominantly focused on individual m6A‐related proteins or specific pathways. The interactions between m6A‐related lncRNAs and immune regulation remain largely unexplored. This study aimed to address this gap by constructing a comprehensive prognostic model that linked m6A‐related lncRNAs to immune function, providing more detailed insights into their clinical significance. In this study, we retrieved *in silico* data and clinical phenotypic information from The Cancer Genome Atlas (TCGA) (http://cancergenome.nih.gov) [17]. Then, we identified the m6A‐related lncRNAs and constructed a prognostic model through bioinformatics analysis. Finally, our findings were further validated by demonstrating that LINC00543 regulated the tumour immune microenvironment in CRC by downregulating STAT1/STAT2 expression.

## 2 Materials and Methods

### 2.1 Dataset Collection and Organisation

As reported in our previous published studies, we obtained the transcriptome profiles, clinical data, and somatic mutation data for 568 CRC tissues from TCGA dataset [18]. We used Perl software to change gene symbols in each pathological dataset to gene IDs, extracted genetic mutation information, and then organised this data into mRNA and lncRNA matrices. Additionally, we analysed the relationship between the expression levels of m6A‐related genes and lncRNAs using the ‘limma’ package in R software. Based on our previous findings, METTL3/14/16, WTAP, VIRMA, ZC3H13, RBM15/15B, YTHDC1/2, YTHDF1/2, YTHDF2, HNRNPC, FMR1, LRPPRC, HNRNPA2B1, JGFBP1/2/3, RBMX, FTO and ALKBH5 were recognised as m6A‐related genes [5, 19, 20, 21].

### 2.2 Construction of an m6A‐Related lncRNA Prognostic Model

We enrolled 540 CRC cases using previously established methods with complete data and pathological information. The CRC cases were randomly divided into a ‘train’ group and ‘test’ group. Then, we conducted Cox regression analysis to identify prognosis‐related lncRNAs. Further, we performed LASSO analysis to construct the m6A‐related lncRNA prognostic model. Moreover, the m6A‐related lncRNA prognostic model was further validated in the ‘test’ group. We also calculated the areas under the receiver‐operating characteristic (ROC) curve in the ‘train’ and ‘test’ groups. The analyses were conducted using the ‘survival’, ‘glmnet’, ‘caret’, ‘survminer’ and ‘timeROC’ packages within R software.

Next, we calculated the C‐index of the m6A‐related lncRNA prognostic model and other clinicopathological features. We also mapped a nomogram based on 1‐, 3‐, and 5‐year survival rates and analysed the data using the ‘survival’, ‘regplot’, ‘rms’, ‘dplyr’, and ‘pec’ packages.

### 2.3 m6A‐Related lncRNA Enrichment Analyses

We divided the tumour samples into high‐risk and low‐risk groups based on the expression levels of m6A‐related lncRNAs. We compared the differentially expressed genes between the two groups. Then, we used R software to perform gene set enrichment analysis (GSEA) to identify immune‐related pathways. A *p* < 0.05 indicated a statistically significant difference.

Furthermore, we compared the differences in immune function between the high‐ and low‐risk groups using the ‘limma’, ‘regplot’, ‘rms’, ‘dplyr’ and ‘pec’ packages in R software. The results were visualised as a heatmap, and a *p* < 0.05 indicated a statistically significant difference.

### 2.4 Cell Line Culture and Transfection

A set of six CRC cell lines, including DLD‐1, HCT‐15, Caco‐2, HT‐29, SW‐480 and SW‐620, was procured besides the non‐malignant colorectal epithelial cell line NCM‐460 (Shanghai, China). These cells were cultivated in RPMI 1640 medium enriched with 10% fetal bovine serum (Gibco, NY, USA), 100 U/mL of penicillin, and 100 μg/mL of streptomycin. The cell lines were cultured in a controlled environment of a humidified chamber at 37°C in the presence of 5% CO_2_ to ensure a sterile environment. In knockdown studies, small interfering RNAs (siRNAs) were used to target the *LINC00543* gene, with the sequences sourced from GenePharma (Jiangsu, China). Lipofectamine 3000 Reagent (Invitrogen) was employed for transfecting these siRNAs into the DLD‐1 and SW‐620 cells. A set of three distinct siRNA constructs, each designed to bind to different regions of the LINC00543 gene (referred to as siRNA‐LINC00543#1, #2 and #3), was used, with a nontargeting siRNA serving as a negative control (si‐NC). The specific sequences for siRNAs were as follows: siRNA‐LINC00543#1: 5′‐GCGAAUCUCUCCAGGGAAUTT‐3′, 5′‐AUUCCCUGGAGAGAUUCGCTT‐3′; siRNA‐LINC00543#2: 5′‐GAGCAUGUGCAAAGAACAATT‐3′, 5′‐UUGUUCUUUGCACAUGCUCTT‐3′; siRNA‐LINC00543#3: 5′‐CCAAGAGAACCAAUCAUAATT‐3′, 5′‐UUAUGAUUGGUUCUCUUGGTT‐3′. Si‐NC: 5′‐UUCUCCGAACGUGUCACGUTT‐3′, 5′‐UGACCUCAACUACAUGGUUTT‐3′.

### 2.5 RNA Isolation and Quantitative Real Time Polymerase Chain Reaction

The RNA was extracted from the samples using the RNAiso Plus reagent (TaKaRa, Shiga, Japan). The integrity and quantity of the RNA were then assessed using the NanoDrop 2000 spectrophotometer (Thermo Fisher Scientific, MA, USA). The PrimeScript RT Master Mix (TaKaRa), which also included a component for the elimination of genomic DNA, was used for converting RNA into complementary DNA (cDNA). The quantitative real time polymerase chain reaction (qRT‐PCR) analysis was conducted on the LightCycler 96 system (Roche Diagnostic, Basel, Switzerland) using SYBR Green (TaKaRa) as the detection dye. The PCR protocol included initial denaturation at 95°C for 30 s, followed by 40 cycles of denaturation at 95°C for 10 s, and annealing/extension at 60°C for 30 s. The expression level of glyceraldehyde 3‐phosphate dehydrogenase was used as the internal control for lncRNA normalisation. The expression level was analysed using the 2^−ΔΔ*Ct*^ method as our previous study [22]. The specific primers for LINC00543 were as follows: forward: CAGACCCAGCAAGGACC; reverse: CCCAGCCATTAGAGCAGA.

### 2.6 Wound Healing and Colony Formation Assay

A wound healing assay was conducted to evaluate the migration capacity of CRC cells. DLD‐1 and SW‐620 cells were seeded in six‐well plates and grown to 90% confluence after transfection with siRNAs targeting the selected gene signature. A sterile 200‐μL pipette tip was used to create a straight scratch in the cell monolayer. The cells were then washed twice with PBS to remove debris and cultured in a serum‐free medium. The images of the wound area were captured at 0 and 48 h using an inverted microscope (Olympus, Tokyo, Japan).

### 2.7 Western Blot Analysis

The cell lysates were prepared using RIPA lysis buffer (Solarbio, Beijing, China) on ice. Sodium dodecyl sulphate–polyacrylamide gel electrophoresis was performed for protein analysis. The isolated proteins were transferred to polyvinylidene difluoride membranes. Subsequently, the membranes were incubated overnight with primary antibodies against STAT1 (1:1000; Abcam, MA, USA), p‐STAT1 (1:1500; Abcam), STAT2 (1:1500; Abcam), p‐STAT1 (1:1000; Abcam), and β‐actin (1:2500; Abcam). The detailed experimental procedures are described in our previously published article [22].

### 2.8 Statistical Analysis

Statistical analyses were conducted, and visualisations were performed using SPSS version 18.0 (SPSS, IL, USA) and Prism version 8.0 (GraphPad, CA, USA). We performed Cox regression to identify prognostic lncRNAs, used LASSO analysis for model construction, and calculated the area under the ROC curve to assess model accuracy. Kaplan–Meier analysis and log‐rank tests were conducted for survival analysis. GSEA was applied to identify immune‐related pathways. The data were presented as means ± standard deviation, and all experiments were performed in triplicate. *p* < 0.05 indicated statistically significant differences.

## 3 Results

### 3.1 Identification of m6A‐Related lncRNAs in CRC

**FIGURE 1:**
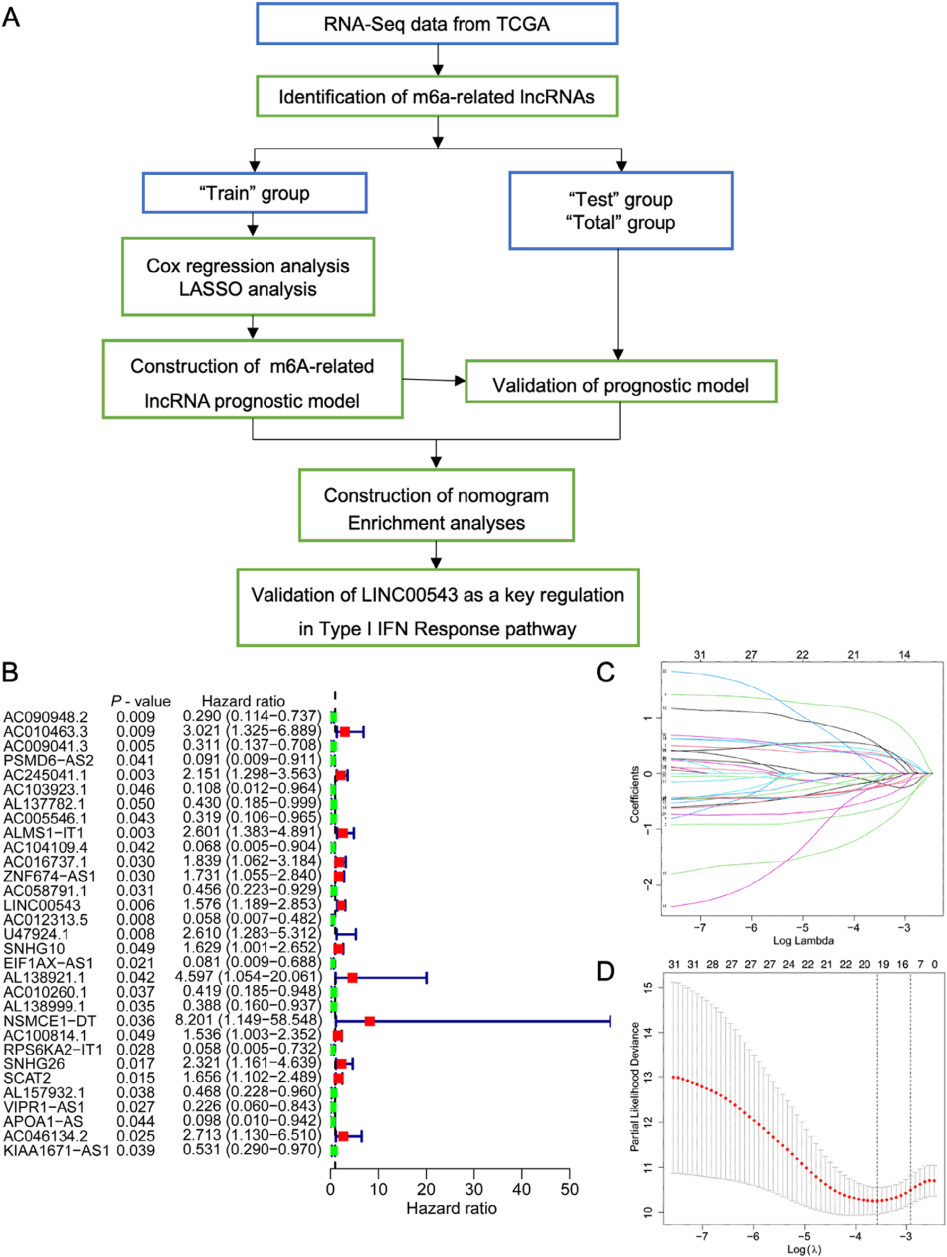
Identification of the m6A‐related lncRNA signature in CRC. (A) Flowchart of the present study. (B) Forest plot illustrating prognostic m6A‐related lncRNAs in the training group. (C) Graphs displaying the LASSO regression coefficients for the m6A‐related lncRNA signature. (D) Cross‐validation plot for the signature.

**FIGURE 2:**
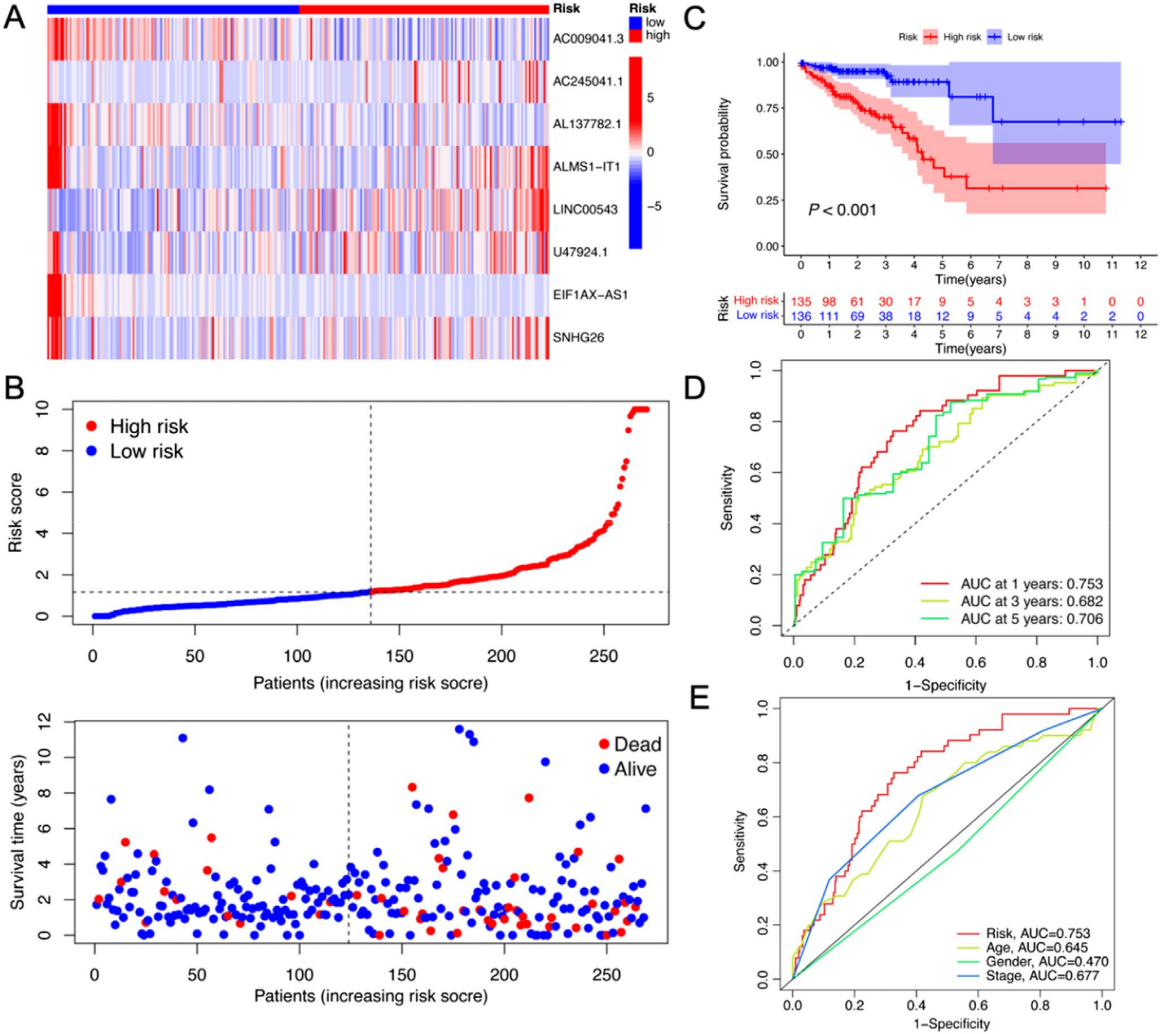
Construction of m6A‐related lncRNA prognostic model. (A) Univariate analysis examining the eight m6A‐related lncRNA signatures in CRC in the training set. (B) Expression levels of m6A‐lncRNA signatures plotted against increasing risk scores in the training set, with the median risk score serving as the threshold to classify samples into ‘high‐risk’ and ‘low‐risk’ categories. The subsequent image illustrates the survival duration and status of patients in relation to the risk score. (C) Kaplan–Meier curve analysis comparing survival outcomes between the ‘high‐risk’ and ‘low‐risk’ groups in the training set. (D) ROC curve evaluation of the prognostic model in training set after 1, 3 and 5 years. (E) ROC curve assessment of the prognostic model in conjunction with age, sex, and stage in the training set at the 1‐year time point.

Initially, we used R software to identify m6A‐related lncRNAs, as outlined in the flowchart shown in Figure 1A. The correspondence between lncRNA and *m6A* genes is visualised and illustrated in Figures S1 and S2. Then, we categorised the CRC samples into ‘test’ and ‘train’ groups and performed C regression (Figure 1B) and LASSO analyses (Figure 1C,D) to construct an m6A‐related lncRNA prognostic model based on the m6A‐related lncRNAs. As shown in Figure 2A, we identified eight m6A‐related lncRNAs (AC009041.3, AC245041.1, AL137782.1, ALMS1‐IT1, LINC00543, U47924.1, EIF1AX‐AS1 and SNHG26) as the lncRNA prognostic model with risk score = (−1.126 × AC009041.3 expression) + (0.574 × AC245041.1) + (−0.79 × AL137782.1 expression) + (1.861 × ALMS1‐IT1expression) + (0.382 × LINC00543 expression) + (0.964 × U47924.1 expression) + (−3.014 × EIF1AX‐AS1 expression) + (−0.897 × SNHG26 expression). Using the derived formula, we computed the risk score for each sample and visualised the prognosis according to these risk scores. The mortality rate was higher in the high‐risk group than in the low‐risk group, indicating a poorer prognosis for those in the high‐risk category (Figure 2B,C).

**FIGURE 3:**
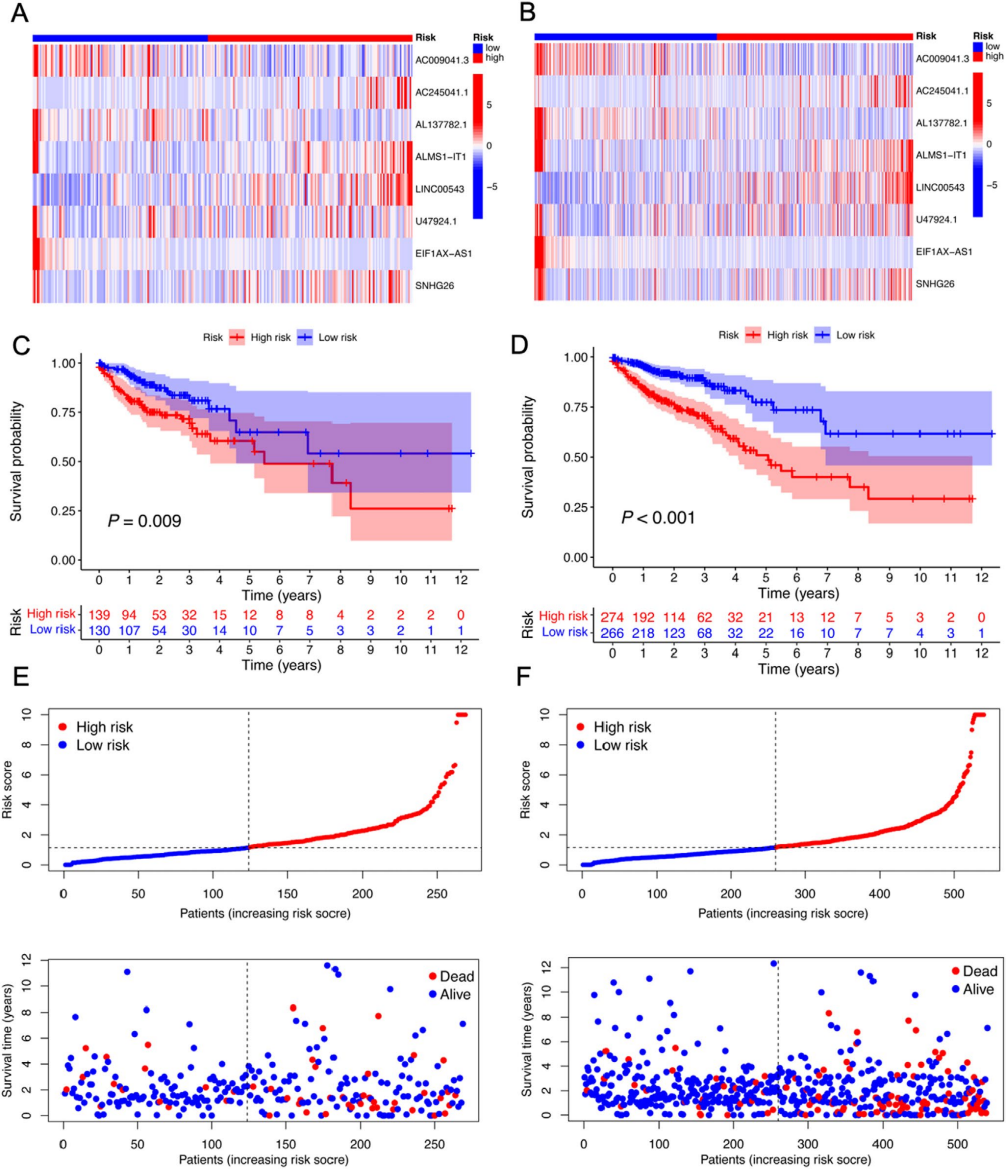
Validation of the m6A‐related lncRNA signatures. (A, B) Expression levels of the m6A‐related lncRNA signatures plotted against increasing risk scores in both the ‘test’ set and the TCGA dataset. The median risk score was used as the threshold to categorise samples into ‘high‐risk’ and ‘low‐risk’ groups. (C, D) Kaplan–Meier curve analysis comparing survival between the ‘high‐risk’ and ‘low‐risk’ groups in the ‘test’ set and TCGA dataset. (E, F) Expression levels of m6A‐lncRNA signatures associated with increasing risk scores in the ‘test’ set and TCGA dataset, where the median risk score served as the cut‐off, dividing samples into ‘high‐risk’ and ‘low‐risk’ groups. The accompanying images depict the survival duration and status of patients with the increase in the risk score.

The area under the curve (AUC) for the ROC curve after 1, 3 and 5 years was calculated to be 0.753, 0.682 and 0.706, respectively, based on the TCGA dataset (Figure 2D,E). Furthermore, the AUC for the prognostic model exceeded that of the TNM staging for the 1‐year prediction (0.753 vs. 0.677). As shown in Figure S1, we performed co‐expression analysis of lncRNAs in the prognostic model and m6A‐associated genes and found a close correlation in their expression. Furthermore, we verified the eight m6A‐related lncRNAs in the ‘test’ set and ‘TCGA’ dataset. As shown in Figure 3A,B, the m6A‐related lncRNAs in the prognostic model were visualised as heatmaps. The results showed that higher risk scores indicated poor prognosis (Figure 3C,D). Furthermore, the mortality rate also increased in both the ‘test’ set and ‘TCGA’ dataset with the increase in risk score (Figure 3E,F).

**FIGURE 4:**
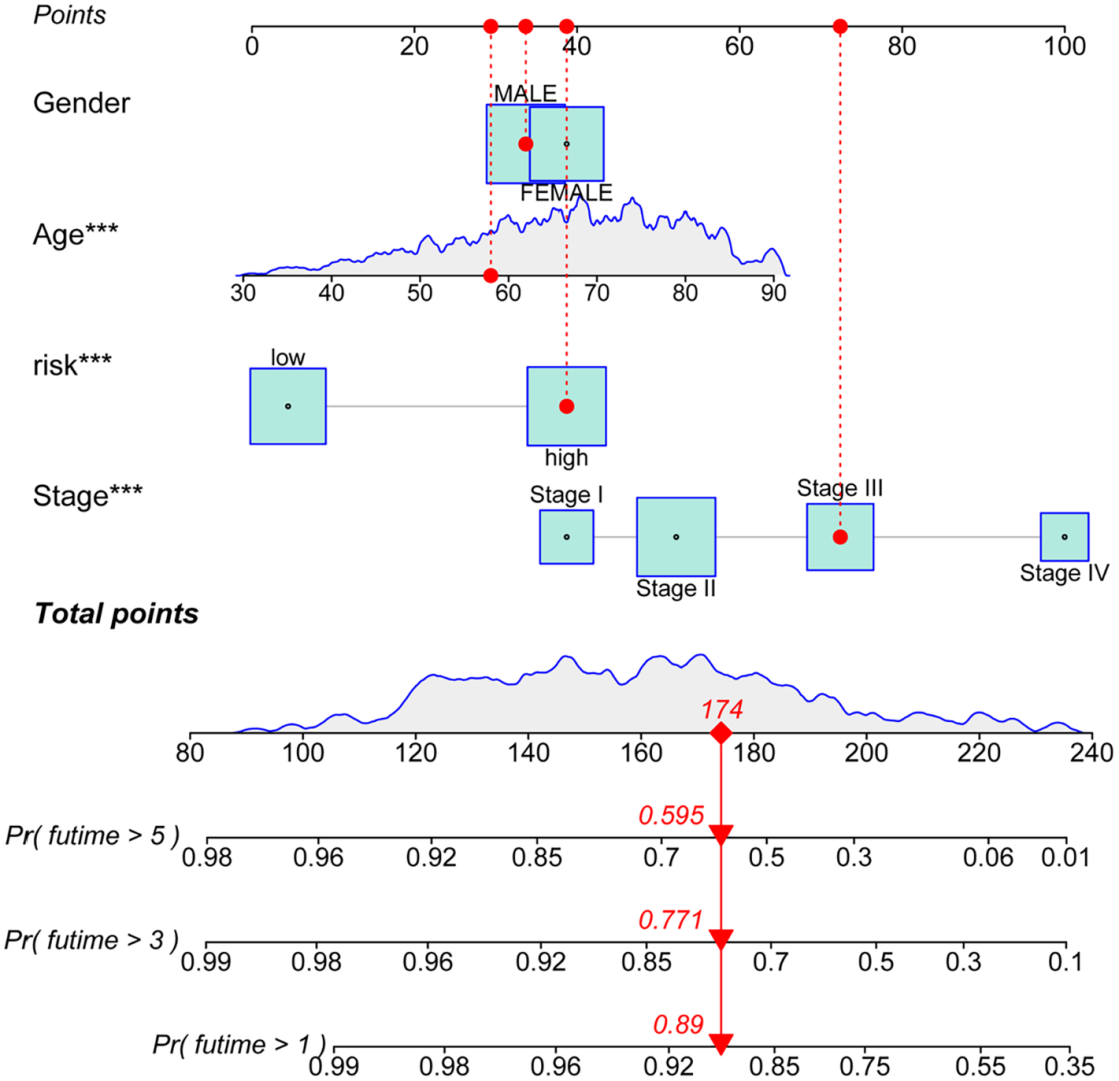
Nomogram of the m6a‐related lncRNA prognostic model. A 58‐year‐old male patient with stage III high‐risk colorectal cancer was used as an example to demonstrate nomograms and their predictions for 1‐, 3‐ and 5‐year survival rates. ***p < 0.001.

**FIGURE 5:**
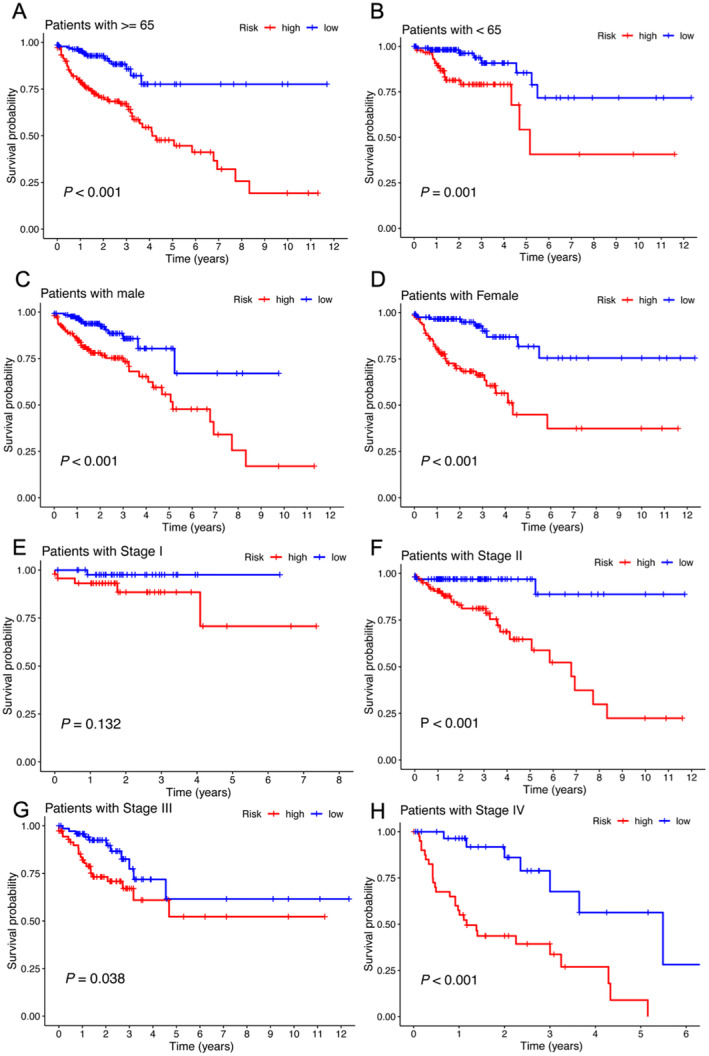
Survival analysis of the m6A‐related lncRNA signature concerning various clinical characteristics. (A, B) Kaplan–Meier curve analysis comparing survival between the ‘high‐risk’ and ‘low‐risk’ groups in the ‘≥ 65 years old’ and ‘< 65 years old’ categories. (C, D) Kaplan–Meier curve analysis assessing the survival differences between the ‘high‐risk’ and ‘low‐risk’ groups among men and women. (E–H) Kaplan–Meier curve analysis examining survival outcomes between the ‘high‐risk’ and ‘low‐risk’ groups across stages I, II, III and IV.

We developed a nomogram using R software to enhance the practicality of the prognostic model for clinical use. As illustrated in Figure 4, factors such as the patient's sex, age, pathological stage, and risk score from the prognostic model were mapped to distinct scores, allowing for prognostic predictions based on these scores. Subsequently, we conducted the Kaplan–Meier curve analysis categorised by age, pTNM stage, and sex to assess the consistency of the m6A‐related lncRNA signature across various pathological characteristics. The findings revealed that the high‐risk group exhibited a poorer prognosis regardless of whether patients were younger or older than 65 years (Figure 5A,B). Consistent results were also observed across both male and female patients, as well as in the Stage I to IV groups (Figure 5C–H).

### 3.2 Immune Function Enrichment by m6A‐Related lncRNAs

**FIGURE 6:**
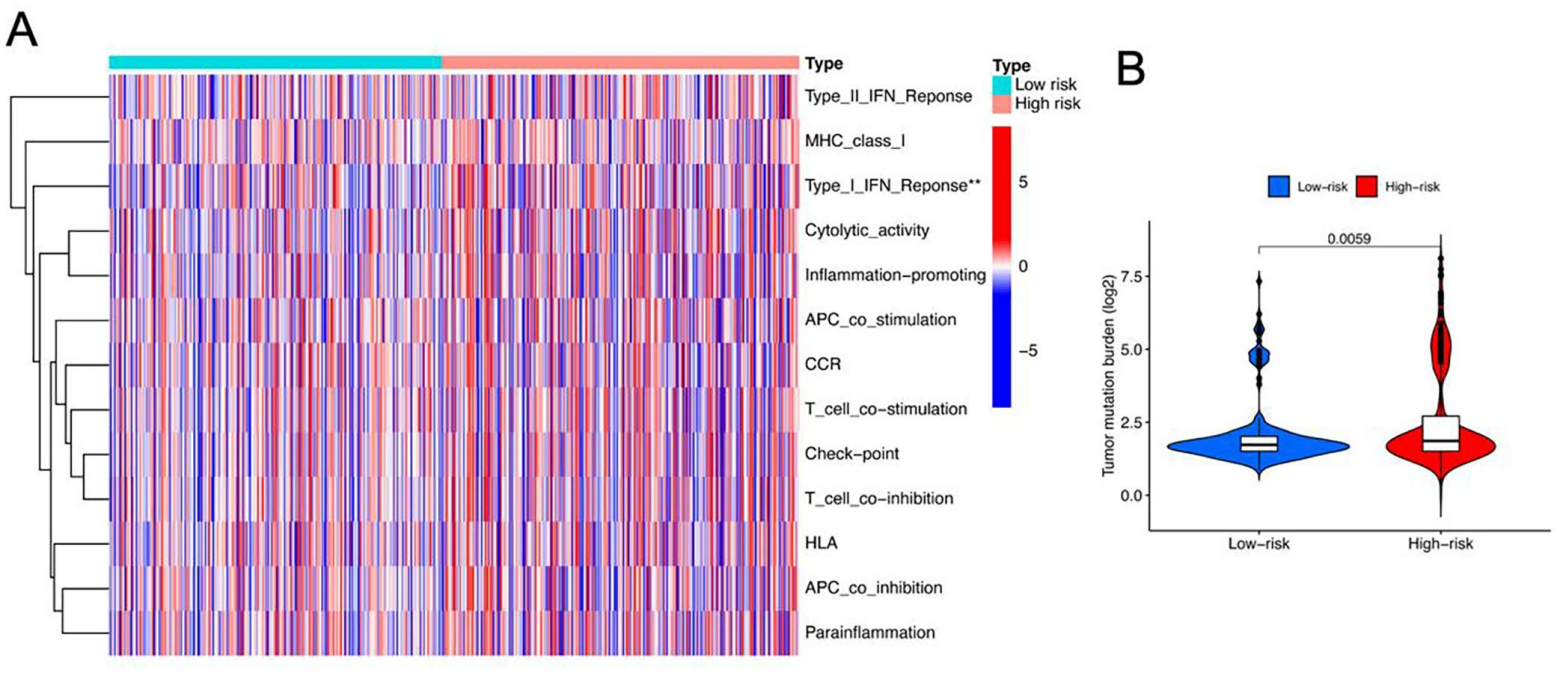
Immune functional enrichment of m6A‐related lncRNA signature. (A) Pathways differentially enriched between the ‘high‐risk’ and ‘low‐risk’ groups. (B) Comparison of tumour mutation burden between the ‘high‐risk’ and ‘low‐risk’ groups. **p < 0.01.

We conducted GSEA to examine immune function disparities between high‐risk and low‐risk groups so as to clarify the role of m6A‐related lncRNAs in the immune regulation of CRC. As depicted in Figure 6A, the notable differences between the two groups included the type I interferon (IFN) response and cytolytic activity. Additionally, the high‐risk group exhibited an increased tumour mutation burden (Figure 6B).

### 3.3 LINC00543 Promoted Type I IFN Response in CRC

**FIGURE 7:**
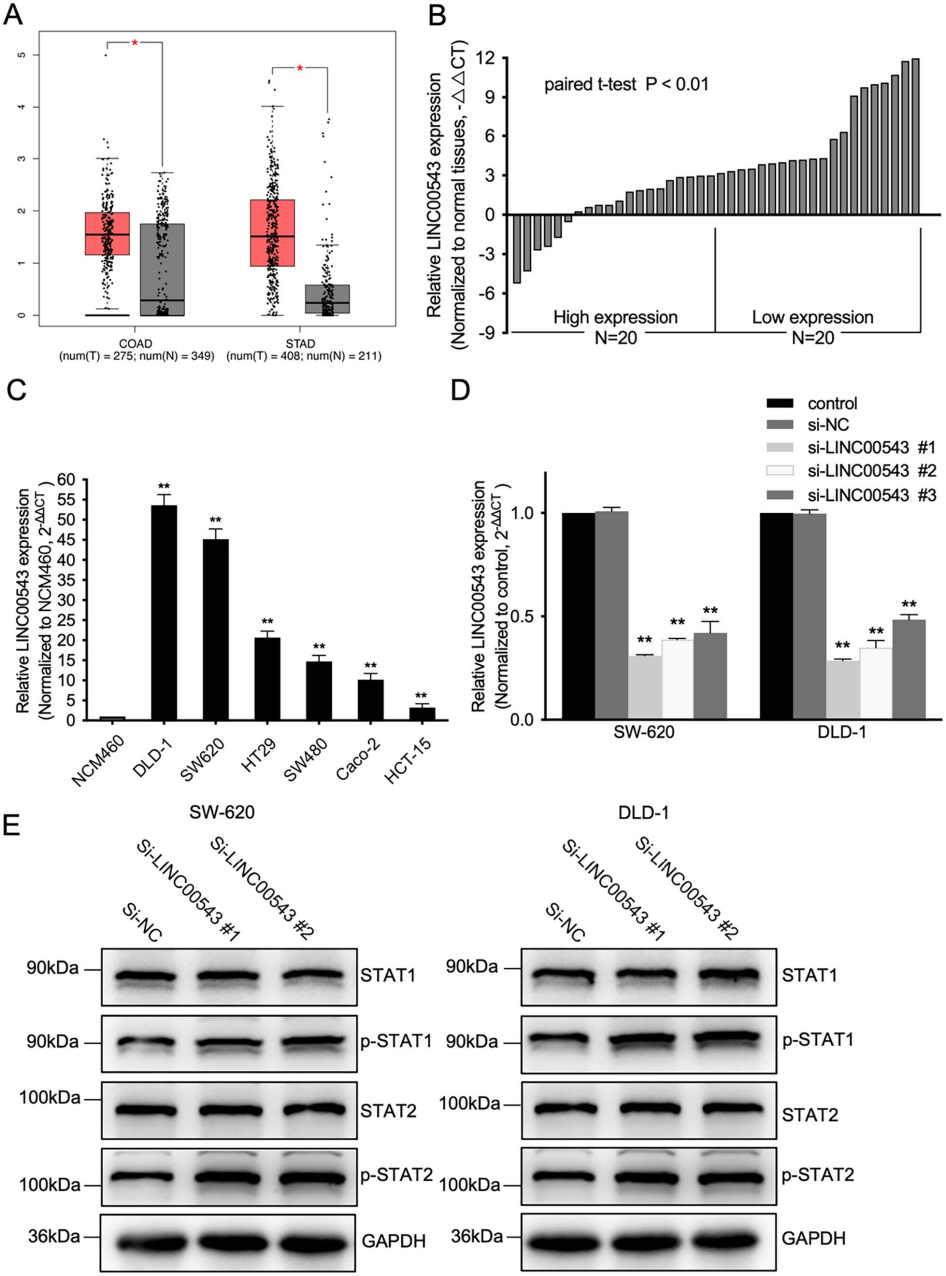
LINC00543 was highly expressed in CRC tissues and inhibited the type I IFN response signalling pathway. (A) Results of the analysis obtained from the Gene Expression Profiling Interactive Analysis website. (B, C) qRT‐PCR experiments demonstrated that the LINC00543 expression was significantly upregulated in CRC tissues and cell lines. (D) qRT‐PCR experiments validated that siLINC00543 effectively reduced the expression of LINC00543 in the DLD‐1 cell line. (E) Protein levels of STAT1 and STAT2 in the SW‐620 and DLD‐1 cell lines, treated with either si‐NC or si‐LINC00543, were assessed using Western blotting. *p < 0.05; **p < 0.01.

When comparing the previously constructed prognostic model with the current prognostic model, we noted the presence of LINC00543 in both prognostic models [18]. In addition, the function and mechanism of lncRNA LINC00543 in CRC remain inadequately understood. Our analysis of CRC samples from the TCGA database revealed significant overexpression of LINC00543 in cancerous tissues (Figure 7A). We conducted qRT‐PCR to measure the expression levels of LINC00543 in 40 CRC samples and their matched noncancerous counterparts. The qRT‐PCR results aligned with the data from the TCGA database (Figure 7B).

We assessed the expression levels of LINC00543 in various CRC cell lines to investigate its role. As shown in Figure 7C, LINC00543 was overexpressed in the DLD‐1, HCT‐15, Caco‐2, HT‐29, SW‐480 and SW‐620 cell lines compared with NCM‐460 cell line. Given that SW‐620 and DLD‐1 exhibited the highest expression levels, we selected these two cell lines for further experiments. We knocked down LINC00543 by transfecting cells with three different siRNAs. Further, we conducted qRT‐PCR 48 h post‐transfection, which confirmed effective suppression of LINC00543 using siRNA #1 and #2 (Figure 7D). The Western blotting was performed to measure the expression levels of STAT1 and STAT2. We observed that knockdown of LINC00543 upregulated STAT1 and STAT2 phosphorylation levels (Figure 7E). As shown in Figure S2B, knockdown of LINC00543 significantly impaired the migration ability of DLD‐1 and SW‐620 cells. The wound closure rate at 48 h was markedly lower in the siRNA‐treated cells than in the control group (*p* < 0.05). Moreover, the number of colonies formed by DLD‐1 and SW‐620 cells after LINC00543 knockdown was significantly reduced (*p* < 0.05) (Figure S2B).

## 4 Discussion

The alterations in m6A modification in CRC have been shown to influence multiple facets of tumour biology, encompassing tumorigenesis, cancer stemness, metastasis and resistance to chemotherapy [5, 6]. Moreover, m6A modification and its cross‐talk with lncRNAs play a vital role in tumorigenesis and can serve as potential biomarkers for CRC prognosis. Our study presents and offers a significant advancement by integrating m6A‐related lncRNAs with immune functional pathways, enhancing the predictive accuracy of conventional TNM staging and other risk factors. This novel approach not only identifies high‐risk patients more effectively but also provides insights into the potential molecular mechanisms underlying CRC prognosis. Therefore, constructing the m6A‐related lncRNA prognostic model is significant for advancing research in epigenetics in CRC.

In this study, we conducted a bioinformatics analysis to identify m6A‐related lncRNAs using high‐throughput data from the TCGA dataset. Consequently, we developed a model incorporating AC009041.3, AC245041.1, AL137782.1, ALMS1‐IT1, LINC00543, U47924.1, EIF1AX‐AS1 and SNHG26. Zhang et al. [23] found that AC009041.3 was a biomarker related to 5‐methylcytosine in CRC. Multiple researchers identified AC245041.1 as a potential immunotherapy target and prognosis signature in colon cancer [24], stomach adenocarcinoma [25] and cutaneous melanoma [26, 27]. Moreover, Zhang et al. [28] indicated that AL137782.1 promoted lung cancer cell migration by upregulating LMO7. ALMS1‐IT1 was also identified as a prognostic marker in head and neck squamous cell carcinoma [29], small cell lung cancer [30] and CRC [31]. Luan et al. revealed that the ALMS1‐IT1/AVL9 axis promoted the malignant progression of lung adenocarcinoma by activating the CDK pathway. LINC00543 was recently found to play a crucial role in the occurrence and development of CRC. Zheng et al. revealed that LINC00543 decreased the maturation of miRNA‐563‐3p, thereby promoting epithelial–mesenchymal transition and remodelling the tumour microenvironment of CRC [32]. Lv et al. [33] identified EIF1AX‐AS1 as a regulator of endometrial cancer cell apoptosis via EIF1AX mRNA stabilization. Furthermore, Wu et al. [34] revealed the upregulation of SNHG26 in gastric cancer, promoting tumorigenesis and metastasis by enhancing c‐Myc translation and activating a positive feedback loop of energy metabolism via the c‐Myc/HK2 pathway.

We categorised the samples into high‐risk and low‐risk groups based on the expression levels of the m6A‐related lncRNA signature, revealing varying prognoses based on different pathological characteristics. The AUC value for the signature was approximately 0.7 after 1, 3 and 5 years, demonstrating the high accuracy of the prognostic model. Additionally, establishing a nomogram enhanced the clinical applicability of the findings from this study.

Our previous study found that LINC00543 was highly expressed in CRC and might impact the immune microenvironment of CRC [18]. As LINC00543 is also present in the prognostic model of this study, this prompted us to further elucidate its role. Functional experiments revealed that LINC00543 significantly promoted CRC cell migration and proliferation, highlighting its potential as a therapeutic target. Through immune function enrichment, we found that m6A‐lncRNA might be associated with the type I IFN response pathway. Consequently, we conducted rescue assays, demonstrating that LINC00543 regulated the expression of STAT1 and STAT2. The dysregulation of both STAT1 and STAT2 has been implicated in various types of cancer, including CRC. Aberrant STAT1 and STAT2 phosphorylation can be activated by IFN signalling, executing the antitumor effects of IFNs and contributing to tumour immune responses [35]. Further studies are required to delineate the functional significance of LINC00543 and its interaction with other genetic elements, providing a comprehensive understanding of its role in CRC.

The present study underscores the crucial role of m6A‐related lncRNAs in CRC prognosis and immune regulation, offering significant clinical implications. The developed prognostic model, based on eight m6A‐related lncRNAs, demonstrates high predictive accuracy and identifies high‐risk patients more effectively than conventional staging methods. Furthermore, the involvement of these lncRNAs, particularly LINC00543, in modulating immune pathways such as the type I IFN response highlights potential therapeutic targets. These findings provide a foundation for integrating epigenetic biomarkers with immunotherapy strategies, paving the way for more personalised and effective CRC treatment approaches.

This study had a few limitations. First, our findings were primarily based on data from the TCGA database, which might have introduced selection bias due to its retrospective nature. Second, although LINC00543 was experimentally validated, the roles of other lncRNAs in the prognostic model remained unverified and warrant further investigation. Integrating single‐cell sequencing and spatial transcriptomics can provide a more detailed understanding of the interplay between m6A‐related lncRNAs and the tumour immune microenvironment. Finally, the model's clinical applicability needs to be confirmed through prospective studies with larger, multicenter cohorts.

## 5 Conclusions

Our study employed a bioinformatics approach to identify an m6A‐related lncRNA prognostic model and uncover its underlying mechanisms by leveraging the associated analytical software and biological experiments. The profound implications of the m6A‐related lncRNA signature in m6A modification suggest that it may serve as a valuable tool for subsequent research and clinical applications, indicating its importance in the broader scope of biological studies.

## Author Contributions

**Peng Jiang:** data curation (supporting), investigation (lead), software (equal). **Mingfei Chu:** data curation (equal), methodology (lead), writing – original draft (supporting), writing – review and editing (supporting). **Yu Liang:** conceptualization (equal), formal analysis (equal), funding acquisition (equal), software (lead), validation (lead), writing – review and editing (equal).

## Conflicts of Interest

The authors declare no conflicts of interest.

## Supporting information


Figures S1–S2.

## Data Availability

The raw data for this study were obtained from the TCGA database (https://portal.gdc.cancer.gov/), which is a publicly available database.
