# Supplementary material for: Identification and Validation of a m6A‐Related Long Noncoding RNA Prognostic Model in Colorectal Cancer
Source: J Cell Mol Med. 2025 Jan 27;29(2):e70376. doi: 10.1111/jcmm.70376 (PMC11770481; doi:10.1111/jcmm.70376)
Supplement: Supplementary file 1 — Figures S1–S2. [file JCMM-29-e70376-s001.docx]

**
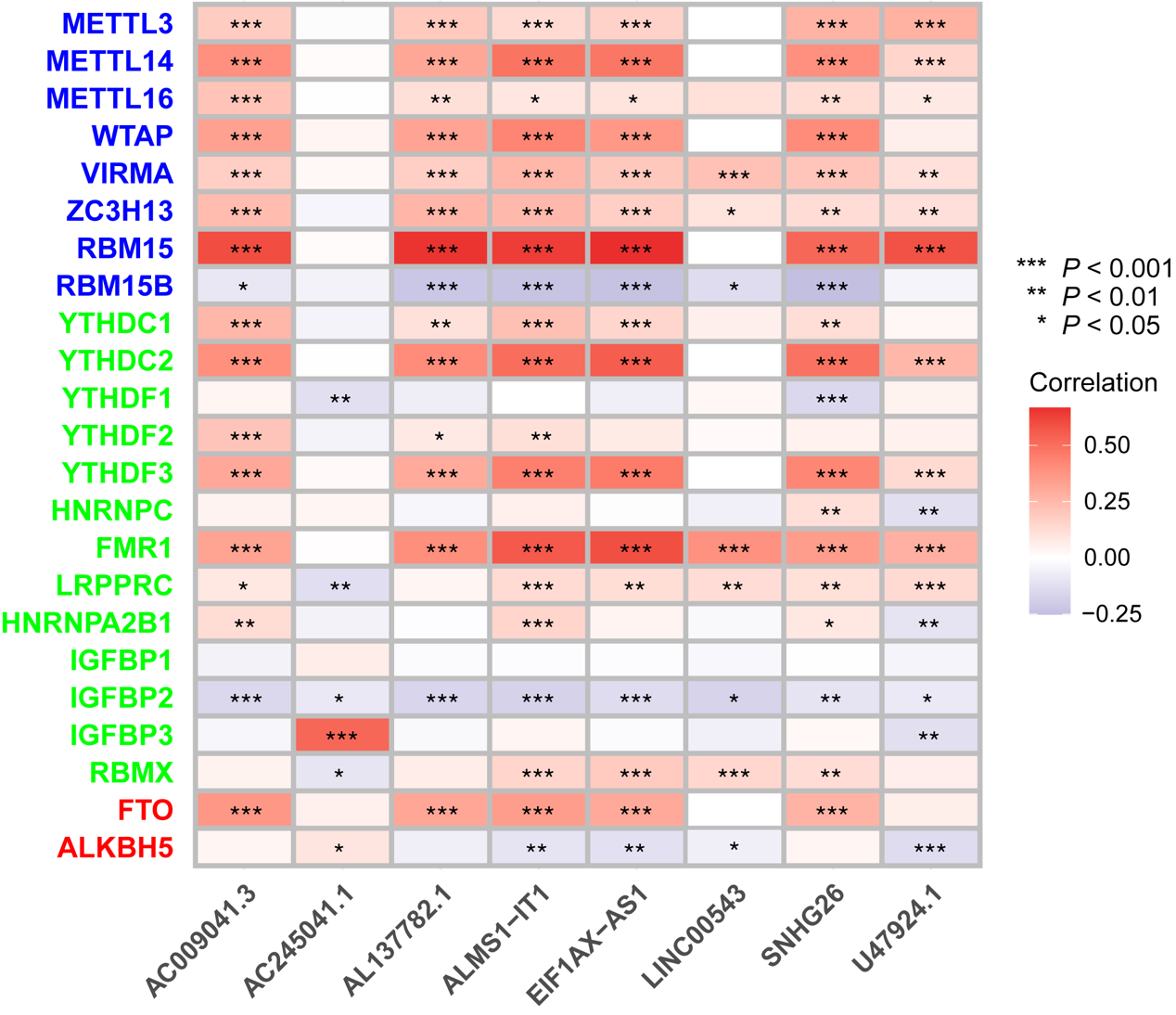
**

**Figure S1. Correlation between m6A-related lncRNA signatures and m6A-related genes.** Red indicates a positive correlation, whereas blue signifies a negative correlation.

**
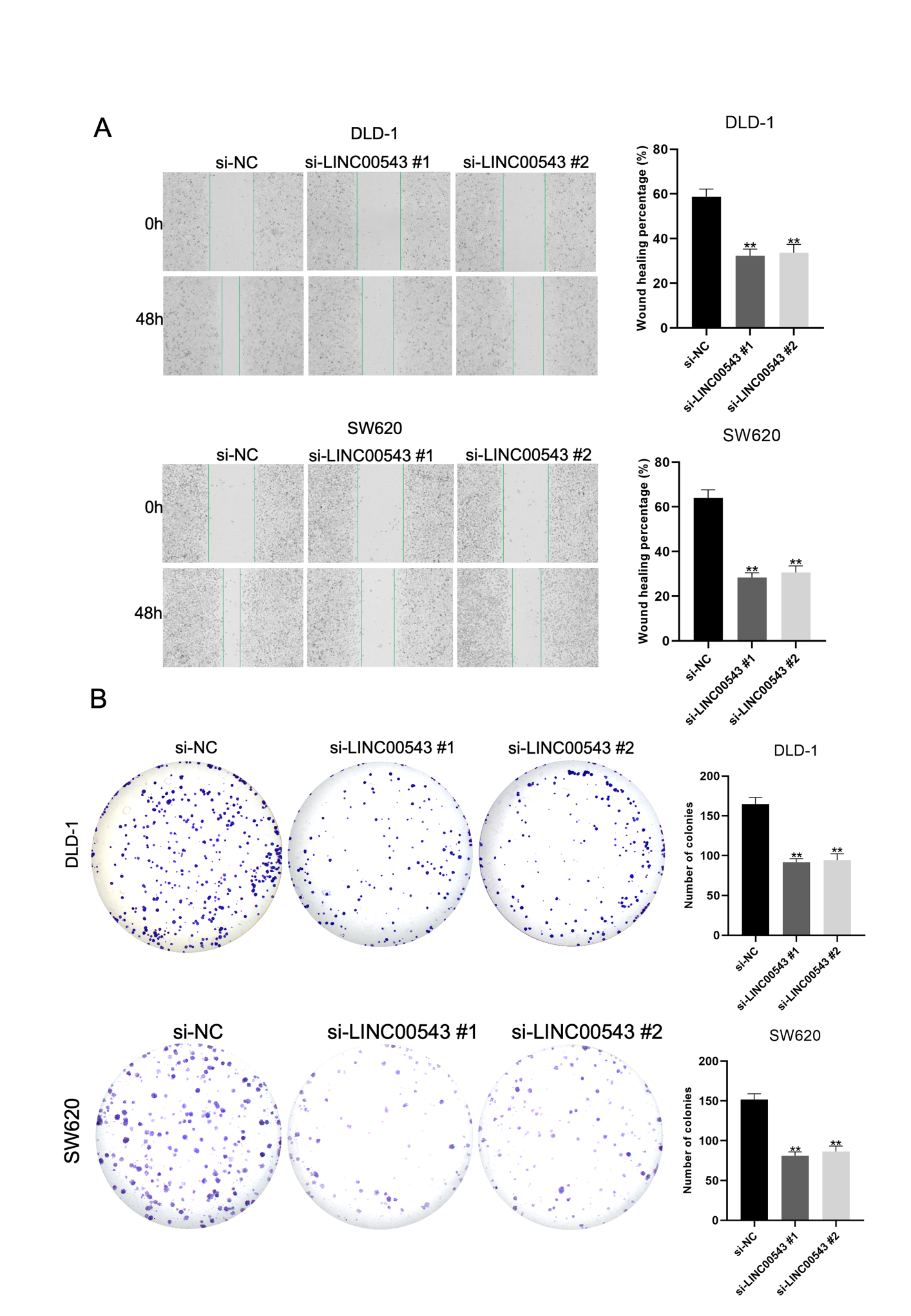
**

**Fig S2. Silencing LINC00543 can inhibite biological function of CRC cell lines.** (A) Transfecting si-NC or si-LINC00543 in DLD-1 and SW620 to generate wound healing experiments to explore the cell migration abilities. (B) Transfected DLD-1 and SW620 cells by si-NC or si-LINC00543 were used to conduct colony formation experiments to explore the cell proliferation abilities. *Means P < 0.05, **Means P < 0.01.
